# Making gametes from pluripotent stem cells – a promising role for very small embryonic-like stem cells

**DOI:** 10.1186/1477-7827-12-114

**Published:** 2014-11-24

**Authors:** Deepa Bhartiya, Indira Hinduja, Hiren Patel, Rashmi Bhilawadikar

**Affiliations:** Stem Cell Biology Department, National Institute for Research in Reproductive Health (ICMR), Mumbai, 400 012 India; Hinduja IVF Centre, PD Hinduja Hospital and Medical Research Centre, Veer Savarkar Marg, Mumbai, 400 016 India

**Keywords:** PGCs, Germ cells, Gametes, Sperm, Oocyte, ES cells, iPS cells, VSELs, Infertility, Assisted reproduction

## Abstract

The urge to have one’s own biological child supersedes any desire in life. Several options have been used to obtain gametes including pluripotent stem cells (embryonic ES and induced pluripotent iPS stem cells); gonadal stem cells (spermatogonial SSCs, ovarian OSCs stem cells), bone marrow, mesenchymal cells and fetal skin. However, the field poses a huge challenge including inefficient existing protocols for differentiation, epigenetic and genetic changes associated with extensive *in vitro* manipulation and also ethical/regulatory constraints. A tremendous leap in the field occurred using mouse ES and iPS cells wherein they were first differentiated into epiblast-like cells and then primordial germ cell-like cells. These on further development produced sperm, oocytes and live offspring (had associated genetic problems). Evidently differentiating pluripotent stem cells into primordial germ cells (PGCs) remains a major bottleneck. Against this backdrop, we propose that ***a novel population of pluripotent stem cells termed very small embryonic*-*like stem cells (VSELs) may serve as an alternative, potential source of autologus gametes, keeping in mind that they are indeed PGCs surviving in adult mammalian ovaries and testes****.* Both VSELs and PGCs are pluripotent, relatively quiescent because of epigenetic modifications of parentally imprinted genes loci like Igf2-H19 and KCNQ1p57, share several markers like Stella, Fragilis, Mvh, Dppa2, Dppa4, Sall4, Blimp1 and functional receptors. VSELs are localized in the basement membrane of seminiferous tubules in testis and in the ovary surface epithelium. Ovarian stem cells from mouse, rabbit, sheep, marmoset and humans (menopausal women and those with premature ovarian failure) spontaneously differentiate into oocyte-like structures *in vitro* with no additional requirement of growth factors. Thus a more pragmatic option to obtain autologus gametes may be the pluripotent VSELs and if we could manipulate them *in vivo* – existing ethical and epigenetic/genetic concerns associated with *in vitro* culture may also be minimized. The field of oncofertility may undergo a sea-change and existing strategies of cryopreservation of gametes and gonadal tissue for fertility preservation in cancer patients will necessitate a revision. However, first the scientific community needs to arrive at a consensus about VSELs in the gonads and then work towards exploiting their potential.

## Background

Gametes derived from pluripotent stem cells may provide potential reproductive options to individuals who are rendered infertile due to injuries, exposure to toxicants or immune-suppressive treatments, in cases with gonadal insufficiency due to premature ovarian failure or azoospermia, reproductive aging and idiopathic cases of poor gametes quality and IVF failure. These artificial gametes derived from stem cells may also serve as an invaluable model system to study both genetic and epigenetic programming of germ cells development *in vivo* and also help obtain better insights into causes for idiopathic cases of infertility. Premature ovarian failure (POF) is a heterogeneous disorder that occurs at the frequency of less than 1% in women less than 40 years of age. Besides genetic basis and autoimmune etiologies, POF is caused by surgical removal of ovaries for conditions such as severe endometriosis, cancer and also as a side effect of oncotherapy for various non-gynecological malignancies. Similarly, besides a genetic basis, azoospermia in men occurs as a side effect of oncotherapy or infections. The option to preserve fertility prior to oncotherapy by way of cryopreservation of gametes or embryos is not yet widely available in several countries and also not useful to young pre-pubertal cancer patients due to non-availability of gametes. Women willingly go through 6–7 failed IVF cycles with a hope to become pregnant. However, assisted reproductive technologies of IVF and ICSI fail to benefit 30% of couples diagnosed with unexplained infertility and in cases where patients are entirely devoid of viable gametes. Donor gametes or adoption are available options however, the urge to have one’s own biological child supersedes any other desire in life. Recent advances in the field of reproductive medicine are focused on exploiting pluripotent stem cells to differentiate into gametes with a hope to deal with infertility.

First human pluripotent embryonic stem (hES) cell lines were reported more than 15 years ago [1] but their induction into gametes remains highly inefficient till date. A recent 2014 Views and Reviews section in Fertility and Sterility was dedicated to stem cells, their differentiation into germ cells and the related efforts towards translation. To summarize it is still a long way before realizing clinical potential of stem cells to make gametes for reproductive medicine [2]. We encourage the readers to refer these publications for latest update in the field [3–7]. Our review provides an altogether a different perspective to overcome existing hurdles to obtain gametes from stem cells. We put forth our case in favor of VSELs as an alternative source of pluripotent stem cells to obtain gametes.

### Pluripotent stem cells differentiation into gametes – recent advances

**Figure 1:**
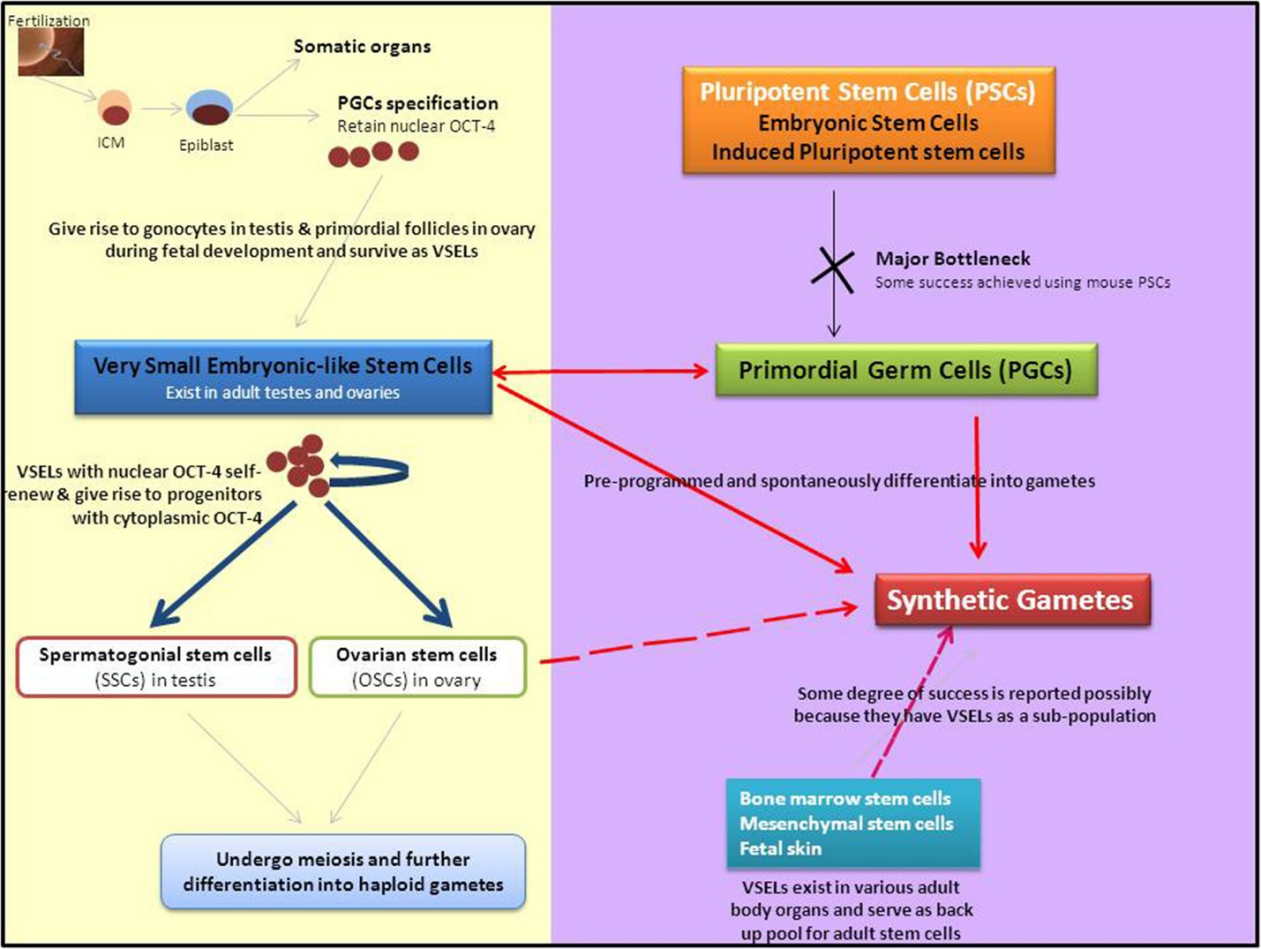
**Left yellow panel depicts event that occur naturally.** Right purple panel represents human efforts to make synthetic gametes. Fertilization of gametes results in a blastocyst with inner cell mass (ICM) which comprises of pluripotent cells (grown in vitro as ES cells) and further develops into a epiblast-stage embryo where specification into somatic cells and primordial germ cells (PGCs) occurs. PGCs are pluripotent, express nuclear OCT-4, differentiate into gonocytes in testes and primordial follicles in ovaries (please refer to the main text for greater details) and persist in adult gonads as pluripotent, nuclear OCT-4 positive VSELs. Thus in addition to SSCs and OSCs in testes and ovaries [42], VSELs also exist [48] as reviewed recently. VSELs self-renew and give rise to progenitors (SSCs in testis and OSCs in ovary) which undergo clonal expansion, meiosis and further differentiation into gametes. Solid blue arrows represent asymmetric cell division of VSELs [48]. Differentiation of ES and iPS cells into synthetic gametes is a distant dream as they do not efficiently differentiate into PGCs. VSELs and OSCs spontaneously differentiate into oocyte-like structures *in vitro*[43, 63, 74–76, 78, 79] as they are indeed PGCs that survive into adulthood. Limited success has been achieved using bone marrow [27–29], fetal skin [30] and mesenchymal cells [31–33] possibly because they have VSELs present as a sub-group. Please note that brown color in the yellow panel represents pluripotent nuclear OCT-4 positive cells.

A careful review of published literature shows that a group from Japan, including Prof. Hayashi and Prof. Saitou has achieved major progress in the field of generating gametes from mouse pluripotent stem cells (mES/iPS cells). In 2011 they published in *Cell* that it is possible to obtain live pups from sperm derived from pluripotent stem cells (ES or iPS cells) [8]. In 2012 they published in *Science* that following a similar strategy, offspring are obtained from oocytes derived from ES or iPS cells [9]. In 2013, they have published their detailed protocols in Nature Protocols describing the method to generate eggs starting with mouse ES cells and iPS cells [10]. Basic reasoning that led to this remarkable success was that it is important to recapitulate *in vitro* what happens *in vivo* during early embryo development. Two main strategies that have been used in the past to induce germ cells from pluripotent stem cells (PSCs) include (i) spontaneous differentiation of PSCs to make embryoid bodies (EBs), isolate cells expressing germ cell markers for further manipulation and (ii) to use mouse epiblast stem cell lines to obtain germ cells. Both these approaches, although provide proof of concept that it may be possible to differentiate PSCs into germ cells, remain highly inefficient. Primordial germ cells (PGCs) are available in very few numbers and are relatively quiescent and thus the embryonic germ cell lines derived from them [11] have shown limited long-term proliferation potential [12]. Thus Hayashi’s group carried out experiments to first differentiate PSCs into epiblast-like cells and then induced them into PGC-like cells (PGCLCs). They demonstrate that once PGCLCs are obtained, it is possible to transplant them into testis/ovary to enable their further differentiation into sperm or oocytes respectively resulting in offspring. It is important to note that in both the publications, Hyashi et al. [8, 9] have reported existence of genetic anomalies in the offspring. When PSCs were induced to undergo spermatogenesis, some of the offspring underwent premature deaths because of tumors around the neck region. Similarly reduced number of pups were obtained from PSCs (3.9%) compared to those obtained by transplanting E12.5 PGCs (12.7%) or 3 weeks oocytes derived pups (17.3%). Almost half of the PSCs-derived oocytes failed to extrude second polar body resulting in 3PN zygotes. This is not surprising since extended cultures of ES/iPS cells are bound to result in the acquisition of genetic and epigenetic alterations during *in vitro* culture and parallel studies in humans remain a distant dream [2, 13]. Besides them, few other groups have also reported that PGCs have the ability to undergo gametogenesis when transplanted in adult tissues. Chuma et al. [14] transplanted PGCs in testis and obtained mature sperm whereas Matoba et al. [15] reported that PGCs isolated from E12.5 male fetus under the kidney capsule yield spermatids. Both these groups reported birth of healthy offspring. Similarly Matoba et al. [15] and Hashimoto et al. [16] reported that PGCs isolated from female fetus when transplanted under the ovarian bursa or kidney capsule result in functional eggs. ***It is intriguing to note that offspring born when starting with PGCs are normal compared to when starting with ES/iPS cells*.** Hayashi et al. [17] reviewed recent advances towards obtaining human gametes to treat infertility. They highlighted the existing hurdles in the existing differentiation protocols and discuss alternative use of germline stem cells (SSCs or OSCs) as a source to produce synthetic gametes (Figure 1). It may also be possible to obtain germ cells by transdifferentiation of somatic cells e.g. bone marrow and mesenchymal cells. Efforts are also ongoing to mature the primordial follicles in ovarian cortical tissue which are cryopreserved prior to cancer therapy.

### Differentiation of germline stem cells (SSCs and OSCs) into gametes

Work on spermatogonial stem cells (SSCs) has progressed and recent reports suggest that it may be possible to expand SSCs (around 0.03% of all testicular cells) *in vitro* in mice [18] and also in men [19]. However, on transplantation –these cells are able to colonize but differentiation remains inefficient. Recent success was reported by Hermann et al. [20] who obtained functional sperm after autologus SSCs transplantation in non-human primates which after IVF also resulted in the formation of blastocysts. However lot more work needs to be done before it can reach the clinic and for more reading in this area readers may refer to recent reviews [21, 22]. Tilly’s group has made significant contributions to the field of ovarian stem cells (OSCs) since their first landmark paper challenging the basic dogma that females are born with fixed number of eggs [23]. OSCs are localized in the ovary surface epithelium and can be isolated from the ovarian cortex, expanded in culture and later transplantation in adult mice - they differentiate into functional eggs and result in offspring [24]. Recently the same group isolated human OSCs, injected in human cortical tissue and on transplantation in immuno-deficient mice demonstrated follicle formation [25]. Several groups are working extensively to mature primordial follicles from cortical tissue slices which include techniques like *in vitro* growth and *in vitro* maturation however challenges remain to be overcome and to develop a perfect culture to obtain a healthy oocyte from primordial follicle [26].

### Trans-differentiation of somatic cells into gametes

Bone marrow has been reported to be a potential source for female [27] as well as male [28] germ cells. Kashani et al. [29] showed that retinoic acid can induce differentiation of mouse bone marrow stem cells into male germ cells. This concept of transdifferentiation of somatic cells into germ cells is intriguing and Dyce et al. [30] were recently able to differentiate both male and female porcine skin fibroblasts to yield oocyte-like cells but more work needs to be undertaken to obtain functional oocytes. Similarly mesenchymal cells have also been proposed to transdifferentiate into germ cells [31–33]. However, the field remains controversial since we and others have reported that indeed ***bone marrow*** [34, 35] ***as well as MSCs*** [36] ***have a sub-group of pluripotent very small embryonic-like stem cells (VSELs)*** which could possibly be responsible for the observations made by various groups (Figure 1). Liu et al. [37] reported that MSCs do not differentiate into sperm but rather heal the damaged testes.

### Primordial germ cells and gametogenesis in mammals

A critical review of literature involving differentiation of PSCs into gametes *in vitro* shows that the crucial step is to differentiate PSCs into PGCs. This has remained a major bottleneck. ***The PGCs appear to be pre-programmed and easily differentiate into gametes*** (Figure 1). This section reviews available understanding as to how the PGCs develop and lead to formation of gametes. Early embryonic development and differentiation of germ cells from the PGCs has been nicely reviewed recently [38, 39]. PGCs arise in proximal epiblast on E7.5 in mice, migrate along the dorsal mesentery- through the aorta-gonad-mesonephros (AGM) region to settle in the gonadal ridge and proliferate in large numbers (from 150 cells on E8.5 to approximately 25,000 cells at E13.5). There exists an intriguing overlap between PGCs migration along the dorsal mesentery and primitive hematopoiesis which is initiated at about the same time in the AGM [40]. Being pluripotent, PGCs are capable of giving rise to both the germ cells as well as the cells of hematopoietic system. By E13.5, PGCs within the genital ridge cease dividing; those in the female enter meiosis and those in the male undergo mitotic arrest. By E15.5, oogonia are formed in females whereas in males they are termed gonocytes. At birth, the gonocytes undergo rapid proliferation to form spermatogonia that further proliferate and differentiate into spermatocytes and undergo meiosis to form sperm. Small proportion of spermatogonial stem cells (with ability to self-renew and further differentiate into sperm) survive in the testis throughout life. Simultaneously, in females the oogonia further differentiate and assemble as primordial follicles during perinatal period and a female has fixed number of follicles which later mature after puberty under the influence of gonadotropins. PGCs seem to disappear from both ovary and testis after fetal development or during postnatal period. SSCs are the stem cells in the testes whereas existence of ovarian stem cells is still debated. Mounting evidence with seminal contributions of Prof Tilly [41, 42], Prof Bukovsky [43] and others is suggestive of existence of stem cells in adult ovary. Data from our lab suggests that **PGCs possibly survive in adult ovary and testis as VSELs** [44–48] similar to that reported in bone marrow and other adult organs [35]. The presence of VSELs in the gonads as well as in the bone marrow may explain the plasticity observed by various groups and ability of bone marrow cells to differentiate into germ cells [27–29].

### Primordial germ cells survive in gonads and other body organs as VSELs in adult mammals including humans

Ratajczak’s group have suggested that the PGCs/their precursors during their migration not only migrate to the gonadal ridges but indeed settle down in various adult organs and serve as a back-up pool of pluripotent stem cells to give rise to tissue specific stem cells to maintain homeostasis [35, 49, 50]. Owing to their small size (3–6 μm) and because they express long telomeres and pluripotent markers [Oct-4, Nanog, Rex-1, SSEA-1 (mice) and SSEA-4 (humans)], these cells are termed very small embryonic-like stem cells (VSELs). VSELs can be sorted as Sca + LIN-CD45- in mice and as CD133 + LIN-CD45- in humans. Similar to ES cells, VSELs also are positive for alkaline phosphatase, have a distinct spherical shape with a large nucleus surrounded by a thin rim of cytoplasm and high nucleo-cytoplasmic ratio. Shin et al. [51] reported that mouse bone marrow VSELs have transcriptionally active chromatin structures for both Oct-4 and Nanog promoters. Their pluripotent state is shown by their ability to self-renew and differentiate *in vitro* into all three germ layers in both mice [52] and humans [53]. VSELs get mobilized in circulation in response to injury [52, 54–58] to regenerate damaged tissues and also in response to G-CSF treatment [59].

After gastrulation, majority of epiblast stem cells lose expression of pluripotency transcription factors and further develop into somatic organs whereas the pluripotency markers are selectively expressed in the PGCs (Figure 1). Various evidence to suggest that VSELs which exist in adult body organs could possibly be the PGCs or their precursors is summarized in Table 1 and has been elaborately studied by Ratajczak’s group [35, 40, 60, 61]. This is supported by (i) both PGCs and VSELs are pluripotent and relatively quiescent in nature (ii) quiescent nature of both PGCs and VSELs is due to similar epigenetic modification of paternally imprinted genes like Igf2-H19 and KCNK1p57 (iii) both express Stella, Fragilis, Blimp1, Mvh (iv) late migrating PGCs specific markers including Mvh, Dppa 2, Dppa4, Sall4 are also expressed by VSELs. VSELs also express several miRNAs that attenuate Igf-1/Igf-2 signaling in these cells (mir681, mir470, mir669b) as well as up regulate expression of p57 (mir25.1, mir19b, mir92). VSELs have recently been also shown to express functional receptors for genes involved in PGCs specification into gametes. Based on the developmental origin of VSELs, their proliferation, like PGCs, is controlled by the DNA methylation state of some of the developmentally crucial imprinted genes (e.g. *H19, Igf2,* and *Rasgrf1*). During the ageing process, proliferation-repressive epigenetic marks progressively disappear, resulting in the increased sensitivity to Ins/Igf signaling and thus depletion of VSELs [62]. A direct developmental link between PGCs and hematopoiesis was recently discussed by Kucia et al. [40]. A lot of overlap exists among chromosomal aberrations between germline tumors and leukemias or lymphomas suggesting their clonal origin from common precursor VSELs. Thus it is likely that ***a common population of VSELs exists in adults that undergoes hematopoiesis in bone marrow and gametogenesis in the gonads***. It is time to think beyond the existing paradigm that PGCs migrate only to the gonadal ridge and give rise to germ cells – rather they possibly migrate and settle in various adult organs and survive throughout life serving as a backup pool for tissue committed stem cells.

**Table 1:** **Current understanding and comparison of PGCs with VSELs isolated from mouse bone marrow and adult** 720 **mouse and human ovary and testis**

1	**PGCs versus VSELs isolated from mouse bone marrow [** [40]**,** [50]**,** [59]**]**
	VSELs have been studied in details and compared to PGCs based on available literature but a direct comparison of the two has yet not been made
	**•** PGCs are pluripotent cells derived from epiblast stage embryo whereas VSELs are pluripotent stem cells detected in mouse bone marrow (and other adult body organs). Both have a distinct spherical shape, large nucleus, a thin rim of cytoplasm and long telomeres · VSELs are more differentiated than inner cell mass-derived ESC and share a lot of markers with EpiSC (Gbx2, Fgf5 and Nodal).
	**•** VSELs exhibit similarity in gene expression and epigenetic signatures to epiblast-derived migratory PGCs (but not post-migratory PGCs). However some differences do exist in the gene expression which could be explained by the effect of the niche where they reside (genital ridge for PGCs and bone marrow for VSELs).
	**•** VSELs and the PGCs exhibit similar mechanism of imprinting erasure. Global erasure of parental imprints occurs in PGCs whereas in VSELs erasure mainly on paternally imprinted DMRs (H19-Igf2, RasGRF1) has been reported. DMRs for selected maternally methylated genes (Kcnq1, Igf2R) in PGCs – are hypermethylated in VSELs. In contrast to PGCs, VSELs exhibit an erasure of imprint especially for paternally imprinted DMRs
	**•** Stella promoter in VSELs like in PGCs is partially demethylated and shows transcriptionally active histone modifications (H3Ac and H3K4me3)
	**•** Bone marrow VSELs express at both mRNA and protein levels, genes specific to epiblast (e.g. Stella, Fragilis and Blimp 1) and also those specific to migratory PGCs specification e.g. Dppa2, Dppa4 and Mvh. Both express pluripotent markers including nuclear Oct-4, Nanog, Rex-1, SSEA-1. Both express CXCR4 which is responsible for their migration/mobilization.
	**•** VSELs express several pituitary and gonadal hormone receptors and Sall4 (an early marker shared by germ and hematopoietic cells).
	**•** VSELs similarly like PGCs could be specified to hematopoietic lineage.
2	**Adult mouse and human testis & ovary VSELs compared to bone marrow VSELs** **[** [45]**,** [46]**,** [63]**,** [74]**]**
	**•** Mouse bone marrow VSELs are LIN-/CD45-/SCA-1 +. Similarly VSELs isolated from the mouse ovary and testis are LIN-/CD45-/SCA-1 + .
	**•** Both are very small in size, spherical in shape and have high nucleo- cytoplasmic ratio
	**•** Both express pluripotent markers at mRNA and protein level including nuclear OCT-4, Nanog, Rex-1, SSEA-1 (SSEA-4 in humans)
	**•** Gonadotropin receptors (FSHR) have been reported on both gonadal and bone marrow VSELs
	**•** Human ovarian and testicular VSELs were FACS sorted using SSEA-4 as a marker. Both PGCs and human ovarian and testicular VSELs stain positive for alkaline phosphatase. VSELs express several genes related to pluripotency and self-renewal (POU5F1 (OCT4), SALL4, CDH1, LIN28B, NANOG, SOX2, SOX11, DPPA3 (STELLA), LEFTY1, ZIC3, ZIC5, PRDM14, GAL, PPP1R9A, RNF2, LASS1 (CERS1), SMO, MMP25, GULP1, MLLT4, BMP7, MYBL2, DNMT3B, ZFP42, HESRG, ZSCAN10, TRO, GLI2, FBN3 and DDX11) and PGC related genes (VASA, PRDM1, DPPA3)
3	**VSELs compared to embryonic stem (ES) cells** **[** [47]**,** [59]**,** [70]**,** [74]**]**
	**•** In contrast to ES cells isolated from inner cell mass and iPS cells – VSELs do not form teratomas in SCID mice nor complement blastocyst development. This basic difference between these two populations of pluripotent stem cells is due to novel epigenetic mechanism of imprint erasure on paternally imprinted DMRs (H19-Igf2, RasGRF1) exhibited by VSELs
	**•** VSELs express pluripotent transcripts like ES cells but several folds low expression is reported
	**•** 341 genes were down regulated and 435 genes were up regulated when hES cells were compared to VSELs isolated from human ovary. Interestingly genes like H19 (maternally imprinted gene), VASA (germ cell marker) and PLD6 (required for gametogenesis and meiosis) were up regulated in VSELs compared to hES cells [70].
	**•** ES cells undergo symmetric cell divisions, are immortal *in vitro* and give rise to cells of all three lineages. VSELs remain quiescent and do not readily divide in culture. They self-renew under special conditions and are capable of giving rise to cells of all three lineages e.g. VSELs in ovary surface epithelium respond to FSH, undergo self-renewal and clonal expansion (evidenced by germ cell nest formation) followed by differentiation into oocytes both *in vitro* [70] and *in vivo* [47].
	**•** **Distinct expression profile of VSELs shows that they are more related to PGCs than ES cells and thus have the potential to spontaneously differentiate into gametes.**

VSELs (PGCs) have been reported in adult human [45] and mouse [44, 63] testis. They are localized in the basal seminiferous epithelium of testicular tubules. Similarly they are localized in the adult mouse, rabbit, sheep, marmoset and human ovary surface epithelium [48, 64]. To conclude, in addition to the OSCs reported by Tilly’s group in adult mouse ovary surface epithelium as equivalent to SSCs in the testis [42], we have accumulated evidence for the presence of an additional population of pluripotent stem cells termed VSELs in both adult ovary and testis (Figure 1). VSELs are relatively quiescent and express nuclear OCT-4 along with other pluripotent transcripts whereas the SSCs/OSCs divide rapidly with incomplete cytokinesis and express cytoplasmic OCT-4 [48]. Thus we propose that pluripotent VSELs give rise to the SSCs/OSCs which further differentiate and undergo meiosis to form haploid gametes (Figure 1). This stem cell biology in the gonads is strictly under the control of the somatic microenvironment/niche. With increased age the niche function (source of growth factors and cytokines crucial for stem cells differentiation) gets compromised and perhaps this results in menopause as suggested by others also [65]. Moreover, uncontrolled proliferation of VSELs possibly results in tumors [66, 67]. It is interesting to note that nuclear OCT-4, a marker for VSELs is also being reported as a specific and sensitive marker for testicular tumors [68] and also in ascites fluid of ovarian cancer patients [69].

Thus of the two models proposed by Felici and Barrios [38], we agree with the second model which suggest the existence of a small population of VSELs amongst OSCs/FGSCs in ovary and also a similar sub-population of VSELs exists amongst SSCs in the testes. VSELs undergo characteristic asymmetric cell division wherein they self-renew and also give rise to SSCs/OSCs which undergo rapid symmetric cell division and further meiosis and differentiation to form haploid gametes (Figure 1). We have further shown that these VSELs are indeed responsible for neo-oogenesis and primordial follicle assembly in mice [47], are regulated by FSH [70], form Balbiani bodies, undergo cytoplasmic streaming and germ cell clusters do form during the process in adult ovary [46, 71] as against the recent conclusions made by Lei and Spradling [72]. Parte et al. [46] have reported that ***adult peri-menopausal ovarian VSELs express Stella and Fragilis (specific markers for PGCS) suggesting that the VSELs are indeed the PGCs that survive into adulthood***.

We have further observed that VSELs in the mouse ovary and testis survive chemotherapy (personal observations) in agreement with earlier report in mouse bone marrow after total body irradiation [73]. VSELs exist in otherwise azoospermic testes of survivors of childhood cancer (personal observations) and may also exist in the ovaries which undergo pre-mature failure due to oncotherapy – but are unable to differentiate because the somatic niche gets compromised as a result of oncotherapy. It will indeed be of interest to study whether these persisting VSELs (PGCs) in chemoablated testes and ovaries can spontaneously differentiate into gametes *in vitro*. Anand et al. [74] recently reported that testicular VSELs in mice survive busulphan treatment and readily undergo spermatogenesis when a healthy niche is provided.

Table 1 summarizes the similarity between bone marrow VSELs and PGCs, adult gonadal and bone marrow VSELs and also between VSELs and embryonic stem cells. It is clearly evident that VSELs are pluripotent in nature like ES cells and PGCs but unlike ES cells, express PGCs specific markers and epigenetic profile. Genes like H19 (maternally imprinted gene), VASA (germ cell marker) and PLD6 (required for gametogenesis and meiosis) are up regulated in VSELs compared to hES cells. **This distinct expression profile of VSELs isolated from adult human ovary shows that they are more related to PGCs than ES cells.**

### Ovarian VSELs express FSHR, respond to gonadotropins and undergo neo-oogenesis in adult mouse ovary

Using adult mice, our group has recently documented the effect of ovarian stimulation on the stem cells (VSELs and OSCs) localized in the OSE [47]. Ovaries were studied after 2 and 7 days of treatment with FSH analog (pregnant mare serum gonadotropin PMSG, 5 IU). The changes observed were not related to ovulation since the mice were not administered HCG. We showed that stem cells localized in the OSE respond to PMSG and undergo proliferation, clonal expansion to form germ cell nests, meiosis and differentiate into oocyte-like structures which assemble as primordial follicles. Thus in addition to FSH action on the growing follicles, FSH also has a crucial role in regulating neo-oogenesis in adult ovary from the stem cells localized in the OSE. Detailed studies in sheep show that ovarian stem cells express FSHR, respond to FSH via alternatively spliced FSHR transcript FSHR3 and germ cell nests were observed after 15 hrs of treatment [70]. Kucia et al. [59] also reported presence of pituitary and gonadal hormone receptors on bone marrow VSELs. The VSELs multiply and show enhanced BrdU uptake in response to stimulation by danazol, FSH, LH, PMSG and sex hormones.

### Spontaneous differentiation of ovarian stem cells into oocyte-like structures and parthenotes *in vitro*

Bukovsky et al. [43, 75] first demonstrated differentiation of surface epithelium of post-menopausal human ovary and development into oocytes and blastocysts *in vitro*. Oocyte-like structures were obtained *in vitro* using samples collected from menopausal women as well as those who had premature ovarian failure unlike the conventional IVF procedure where maternal age more than 35 years is considered as a high risk due to the genetic abnormalities. Later Virant-Klun and her group [76–78] reported that very small, embryonic-like, spherical cells could be isolated by OSE scraping of postmenopausal and women with pre-mature ovarian failure. They also reported spontaneous development of oocyte-like structures and parthenogenetic blastocyst-like structures with normal ploidy status. Our group observed that OSE cells from adult rabbits, monkey, sheep and peri-menopausal women (who are otherwise devoid of follicles) when put in culture for three weeks result in spontaneous differentiation of oocyte-like, parthenote-like, embryoid body-like structures and also embryonic stem cell-like colonies whereas epithelial cells attach and transform into a bed of mesenchymal cells possibly by a process of epithelial-mesenchymal transition [63]. We also noted that the presence of germ cell nests, Balbiani body-like structures and cytoplasmic streaming extensively described during fetal ovary development, are indeed well recapitulated during *in vitro* oogenesis in adult human OSE cultures along with characteristic expression of stem/germ cell/oocyte markers [46]. Time lapse imaging of developing oocyte –like cells with distinctly moving cytoplasmic extensions have also been reported by Bukovsky’s group [43, 79].

The striking fact is the spontaneous nature of this kind of differentiation of VSELs into oocyte-like structures. ***No additional growth factors are added to the medium to induce differentiation of oocyte-like structures***. It appears that the VSELs in the OSE scrapings are pre-programmed to differentiate into oocytes. This is indeed facilitated by the epithelial cells which form a bed of fibroblasts and were present in close association with the differentiating stem cells. Similarly the isolated OSCs also undergo spontaneous differentiation into oocytes in culture [25, 43, 80]. Parte et al. [81] have shown that ovarian cortical tissue slices besides being a source of primordial follicles are also an excellent source of stem cells that spontaneously differentiate into oocyte-like structures after 3 weeks culture. Evidently the reason for this spontaneous differentiation of VSELs into oocyte-like structures is that the VSELs closely resemble PGCs (Table 1 and Figure 1).

## Conclusions

It may be possible to obtain human gametes provided efficient and directed differentiation of ES or iPS cells into PGCs is achieved. But this may not be mandatory since emerging literature suggests that PGCs persist as a sub-population of VSELs along with SSCs in testis and OSCs in ovary. Similar to the PGCs, VSELs are quiescent in nature, do not expand in culture like ES or iPS cells and throughout life serve as a backup pool and give rise to SSCs/OSCs which undergo clonal expansion, meiosis and further differentiation to produce haploid gametes. Ovarian VSELs respond to FSH via FSHR3 and spontaneously differentiate into oocyte-like structures *in vitro* during OSE culture. Similar *in vitro* culture studies are ongoing in our lab using testicular VSELs. More studies are required to further substantiate the potential of VSELs and their ability to differentiate into gametes. We propose that rather than the existing concept of *in vitro* differentiation of stem cells into oocytes and sperm for assisted reproduction, it would be ideal to manipulate VSELs that survive oncotherapy *in vivo* to achieve restoration of gonadal function (since they exist in menopausal/ POF ovary and also in azoospermic human testis).

***In contrast to genetically affected offspring born from ES/iPS derived gametes, healthy offspring born starting with OSCs and the oocytes formed after in vitro spontaneous differentiation of ovarian stem cells show normal ploidy status.*** This is evidently because of the similar epigenetic status of PGCs and VSELs which is possibly difficult to be replicated *in vitro* while differentiating ES/iPS cells into PGCs (although some success has been achieved as described above). Scientific community needs to slow down, re-think and make efforts to exploit clinical potential of pluripotent stem cells (VSELs) and progenitors (SSCs and OSCs) which exist in the adult gonads as an alternate option to ES/iPS cells!

### Key messages

-  Current status of making gametes from pluripotent stem cells (ES and iPS) to help infertile couples is highly inefficient and still remains a distant dream
-  Major obstacle in the field is apparently to establish protocols to obtain primordial germ cells (PGCs) from the pluripotent stem cells (ES and iPS) *in vitro.* PGCs are pre-programmed and hence easily and spontaneously differentiate into gametes
-  Published literature is reviewed suggesting that this challenge of making gametes can be easily overcome since PGCs indeed survive in adult human ovaries and testes as very small embryonic-like stem cells (VSELs)
-  VSELs are pluripotent stem cells (surviving PGCs) which exist as a sub-population localized in the adult ovary surface epithelium and in the basement membrane of seminiferous tubules in the testes. They are present in normal adult and aged testes and ovaries (including POF and menopausal ovaries). Moreover VSELs survive oncotherapy because of their quiescent nature.
-  Three weeks culture (simple culture medium with no added growth factors) of ovary surface epithelial cells enriched with VSELs and ovary stem cells (OSCs) spontaneously differentiate into oocyte-like structures - because the gonadal VSELs (PGCs) and OSCs (arise from the VSELs) are pre-programmed to develop into gametes
-  We propose that rather than manipulating gonadal VSELs (PGCs) *in vitro*, a better approach will be to manipulate them *in vivo* to give rise to functional gametes. This approach will give rise to autologus gametes, with no associated ethical/regulatory constraints and epigenetic/genetic issues may not exist by avoiding *in vitro* culture.

## Authors’ information

DB is working on pluripotent stem cells for almost 11 years. IH is leading IVF expert and well understands the need of synthetic gametes by infertile couples. HP is a PhD student at NIRRH and RB works at Hinduja Hospital.

## Abbreviations

ES cells: Embryonic stem cells
FSH: Follicle stimulating hormone
iPS cells: Induced pluripotent stem cells
MSCs: Mesenchymal stem cells
OSCs: Ovarian stem cells
OSE: Ovarian surface epithelial cells
PGCs: Primordial germ cells
POF: Premature ovarian failure
PMSG: Pregnant mare serum gonadotropin
PSCs: Pluripotent stem cells
SSCs: Spermatogonial stem cells
VSELs: Very small embryonic-like stem cells.
